# Prevalence and predictors of PTSD and resilience among Adolescents and Young Adults: Findings from the MoreGoodDays Support Program in Alberta, Canada

**DOI:** 10.1192/j.eurpsy.2024.637

**Published:** 2024-08-27

**Authors:** A. Belinda, R. Shalaby, K. Hay, R. Pattison, E. Eboreime, M. Korthuis, Y. Wei, V. I. O. Agyapong

**Affiliations:** ^1^ University of Alberta; ^2^Kickstand, Edmonton; ^3^Dalhousie University, Halifax; ^4^Glenrose Rehabilitation Hospital Foundation, Edmonton, Canada

## Abstract

**Introduction:**

Adolescents and young adults have particularly been impacted by the COVID-19 pandemic, leading to a rise in the incidence of mental health issues. Increased exposure to traumatic events may lead to decreased resilience and subsequently increased likely PTSD.

**Objectives:**

This study sets out to examine the predictors and prevalence of likely PTSD and determine the level of resilience among adolescents and young adults.

**Methods:**

A cross-sectional study using an online survey questionnaire was adopted to collect sociodemographic and clinical information from the subscribers of MoreGoodDays. The PTSD Checklist Civilian (PCL-C) and the Brief Resilience Scale (BRS was respectively used to assess likely PTSD and resilience Data was analyzed with SPSS version 25 using chi-squared tests and multivariate logistic regression analysis.

**Results:**

343 of MoreGoodDays subscribers who participated in the survey were about 343. Most were female (79.0%), and 13.7% were male. Overall, 95 (45.7%) of respondents had likely PTSD and 109 (51.7%) had likely low resilience. Approximately 176 (51.3%) respondents had received mental health counselling, and 64 (35.4%) expressed the desire to receive mental health counselling. When all other variables are controlled in the regression model, respondents who have received mental health counselling in the past year were 13.7 times more likely to experience likely PTSD (OR = 13.70; 95% CI: 1.23- 142.86) and 15.15 times more likely to experience low resilience than those who did not (OR = 15.15; 95% CI: 1.46- 166.67). Again, those who would like to receive mental health counselling were 20.8 times more likely to experience PTSD than those who did not (OR = 20.76; 95% CI: 2.61- 165.401) and 29.4 times more likely to experience low resilience than those who did not (OR = 29.42; 95% CI: 3.31- 261.445). Finally, those with four or more ACE scores were 6.2 times more likely to experience likely PTSD than those who had zero scores (OR = 6.24; 95% CI: 1.46- 26.67).

**Conclusions:**

MoreGoodDays subscribers were disproportionally affected by likely PTSD and low resilience, reflecting the devastating effect of the COVID-19 pandemic. Increased ACE has been linked to low resilience, which may also lead to a rise in mental health issues. Strategies to promote resilience may reduce the incidence of likely PTSD. Educational institutions may adopt innovative mental health interventions, including psychological interventions such as mobile text technology, to support the mental health of this cohort. Policymakers and government agencies are encouraged to give the mental health of young adults and youth more prominence on their agenda.

**Disclosure of Interest:**

None Declared

